# Bio-Based Polymeric Substrates for Printed Hybrid Electronics

**DOI:** 10.3390/polym14091863

**Published:** 2022-05-02

**Authors:** Enni Luoma, Marja Välimäki, Jyrki Ollila, Kyösti Heikkinen, Kirsi Immonen

**Affiliations:** 1 Sustainable Products and Materials, VTT Technical Research Centre of Finland, Visiokatu 4, 33720 Tampere, Finland; kirsi.immonen@vtt.fi; 2 Digital Technologies, VTT Technical Research Centre of Finland, Kaitoväylä 1, 90570 Oulu, Finland; marja.valimaki@vtt.fi (M.V.); jyrki.ollila@vtt.fi (J.O.); kyosti.heikkinen@vtt.fi (K.H.)

**Keywords:** flexible printed electronics, flexible hybrid electronics, biopolymer films, renewable-based substrate, screen-printing, surface energy

## Abstract

Printed flexible hybrid electronics (FHE) is finding an increasing number of applications in the fields of displays, sensors, actuators and in energy harvesting and storage. The technology involves the printing of conductive and insulating patterns as well as mounting electronic devices and circuits on flexible substrate materials. Typical plastic substrates in use are, for example, non-renewable-based poly(ethylene terephthalate) (PET) or poly(imides) (PI) with high thermal and dimensional stability, solvent resistance and mechanical strength. The aim of this study was to assess whether renewable-based plastic materials can be applied on sheet-to-sheet (S2S) screen-printing of conductive silver patterns. The selected materials were biaxially oriented (BO) bio-based PET (Bio-PET BO), poly(lactic acid) (PLA BO), cellulose acetate propionate (CAP BO) and regenerated cellulose film, NatureFlex™ (Natureflex). The biaxial orientation and annealing improved the mechanical strength of Bio-PET and PLA to the same level as the reference PET (Ref-PET). All renewable-based substrates showed a transparency comparable to the Ref-PET. The printability of silver ink was good with all renewable-based substrates and printed pattern resistance on the same level as Ref-PET. The formation of the printed pattern to the cellulose-based substrates, CAP BO and Natureflex, was very good, showing 10% to 18% lower resistance compared to Ref-PET and obtained among the bio-based substrates the smallest machine and transverse direction deviation in the S2S printing process. The results will open new application possibilities for renewable-based substrates, and also potentially biodegradable solutions enabled by the regenerated cellulose film and PLA.

## 1. Introduction

Flexible thin film electronics have increasing innovative potential in the fields of displays, sensors, actuators, and in energy harvesting and storage. Flexible hybrid electronics’ technology can be characterised as a combination of printing processes and ink chemistry, as well as substrate material engineering for the manufacturing of lightweight electronic components. The technology involves mounting electronic devices and circuits on flexible substrate materials, which are typically polymer films. The target sectors for flexible electronics are especially in health care, automotive, wearables, mobile communications, and human–machine interfaces. Internet-of-Things-based sensing applications are comprised of a large variety of potential user interfaces in sensing, e.g., for logistic operations, diagnostics, and environmental conditions. Additionally, the expectations for the operational conditions and for the lifespan can vary a lot [1,2,3].

Screen-printing is one of the most utilised technologies for manufacturing flexible electronics. It is a fast and versatile process, which can be carried out cost- and material-effectively. Screen-printing pastes have a typical viscosity range of 500–5000 mPas and, compared to other printing techniques, allow higher material thickness, thus being suitable for printing interconnects and passive circuit elements [3].

Screen-printing can be carried out in sheet-to-sheet (S2S) and roll-to-toll (R2R) processes by using a flat-bed or rotary type of screen-printer. In flat-bed screen-printing the substrates are typically sheets and are printed one by one with a planar printing screen, whereas in rotary screen-printing, the printing screen is a rotating cylinder with a fixed position and the printing is usually executed in continuous R2R processing [3,4,5]. It should also be noted that in R2R processing, both the substrate and the printed patterns need to have flexibility.

Metallic particle inks are typically used in screen-printing due to their high conductivity. To attain a high level of conductivity, they typically require curing at elevated temperatures (150–250 °C). This heat treatment removes insulating components, such as stabilising agents and other additives. Silver is the most widely used metal in inks because of its high bulk conductivity and resistance to oxidation, even though silver is an expensive material, resulting in high ink prices [2]. Ordinarily, a high curing temperature of silver inks also puts a limit on plastic substrates suitable for printed electronics, which has also led to developments for conductive ink materials with lower curing temperatures [6,7].

Nowadays, most of the substrate materials in printed electronics are derived from non-renewable fossil raw materials. Examples of substrate materials are poly(imides) (PI), poly(ether ether ketone) (PEEK), poly(ether sulfone) (PES), poly(ether imide) (PEI), poly(ethylene naphthalate) (PEN), and poly(ethylene terephthalate) (PET). Requirements for substrate materials include dimensional stability, thermal stability, solvent resistance, and mechanical strength [1,8]. Furthermore, the thermal management forms a challenge for the devices, and for the polymer substrate. For instance, the thermal management of the printed and hybrid-integrated light-emitting diode (LED) foil can be improved by implementing a special heat management structure, including thermal vias and slugs, to conduct the excess heat through the substrate to the heat sink, as presented in the paper by Keränen et al. [9].

Materials chosen for this study were bio-based or partially bio-based. Bio-based materials decrease dependency on fossil raw materials, as they can be derived from plant-based renewable resources. Plant-based materials also have a lower carbon footprint than materials derived from fossil resources. Their use is in line with the United Nations (UN) Sustainable Goals 12 and 13 and meets the requirements of the European Union (EU) Bioeconomy and EU Plastic strategies [10,11,12]. Globally, increasing use of electronic equipment will also create environmental concerns at the end-of-life, especially if integrated in packaging, textiles, or small gadgets. Biodegradable or compostable biopolymers can offer an alternative end-of-life solution [13]. Besides having an eco-friendly label, the material also has to exhibit sufficient properties, such as heat stability, mechanical strength, and flexibility, to function well in printed electronics applications.

Poly(ethylene terephthalate) (PET) is already used in printed electronics applications. PET is inexpensive and it has good processability for various products. Nowadays, PET is also produced as a partially bio-based resin (bio-PET), containing 30% bio-based ethylene glycol, which reduces its carbon footprint in comparison to fully fossil-based grades. However, there are several potential routes for the production of bio-PET entirely from renewable resources [14]. At the molecular level, the bio-PET is similar to fossil-based PET and is thus categorised as drop-in polymer and as an easy replacement for it. PET is a semi-crystalline clear polymer with a high melting temperature (*T_m_*) (250–260 °C). Typically, PET film processing involves orientation, which generates a semi-crystalline structure via strain-induced crystallisation, improving the mechanical strength and heat resistance properties. The glass transition temperature (*T_g_*) for amorphous PET is 67 °C and 81 °C for crystalline PET [15,16,17].

Poly(lactic acid) (PLA) currently has the biggest production volume of bioplastics and has already found its place in various applications [18]. PLA is manufactured entirely from renewable feedstocks, and it is compostable in industrial composting conditions. It has good mechanical strength and stiffness; however, its brittleness limits some applications. Furthermore, PLA has good processability and a transparent appearance. The properties of PLA are affected by crystallinity. Similar to PET, the crystallinity of PLA can be improved through orientation and annealing. Crystallisation is primarily defined by two stereo-isomeric forms: poly(D-lactide) (PDLA) and poly(L-lactide) (PLLA). To be crystalline, PLA has to be optically pure, containing >90% of PLLA isomer. The melting temperature and glass transition temperature are dependent on the molecular structure being typically between 150–200 °C and 45–65 °C, respectively [8,19]. Castro-Aguirre et al. and Luoma et al. have studied the suitability of cast-extruded PLA films in printed hybrid electronics processing, with promising results [5,20,21].

Cellulose esters have been commercially important polymers since the 18th century, given that they are some of the oldest biopolymers. They are produced using highly purified cellulose and contain several manufacturing steps which make the cost structure of cellulose esters more expensive than oil-based polymers, even though they are very good materials from a performance perspective. Cellulose esters such as cellulose acetate propionate (CAP) or cellulose acetate butyrate (CAB), are the thermoplastic polymers used in film, coatings, inks, moulding, and fibre applications. They typically have a high glass transition temperature and high modulus [22]. Thermoplastic cellulose esters such as CAP typically contain plasticisers to modify the processing properties that also affect other properties such as *T_m_* and *T_g_*. Another way to modify the properties is to control the substitution degree of cellulose hydrogen groups. A typical processing temperature of CAP materials is between 180–230 °C. CAP has *T_g_* in range of 140–150 °C. CAP has high clarity and it has properties similar to cellulose acetate (CA) [23,24,25]. CAP is typically used as a film former in printing inks and overprint varnishes; being soluble in various ink and coating solvents that show compatibility with other resins in these applications [26].

Another cellulose-based substrate with a transparent plastic-like outlook, but without thermoplasticity, is regenerated cellulose, also called Cellophane™ according to the tradename of its first manufacturer, DuPont, in the early stages of the 20th century [27,28]. During the last 20 years, the manufacturing process for regenerated cellulose has improved and the common viscose process is changing to more environmental processes, e.g., those using ionic liquids and other cellulose dissolving processes, which has increased interest in novel uses for regenerated cellulose [29,30]. As such, the regenerated cellulose films are biodegradable, transparent, thermally stable in typical printing process conditions up to 200 °C, and a good oxygen barrier. Cellophane™ has high mechanical strength properties such as tensile strength of 120 MPa and modulus 12 GPa [31]. Its drawbacks include a low water-vapour barrier and it is not heat-sealable, which has led to the development of different coating methods for improving the barrier properties and heat-seal layers [32,33,34]. The use of regenerated cellulose and other cellulose-based materials in printed electronics was reviewed by Brunetti et al. [35] Another cellulose-based, home compostable, and suitable for marine degradation film is NatureFlex^TM^ from Futamura [36]. Its strength and biodegradation properties were studied, for example, by Rapisarda et al. [37].

In this study, different bio-based materials and their performance in the screen-printing process were examined. The main objective was to find more sustainable substrate film materials for printed electronics applications without compromising their performance.

## 2. Materials and Methods

### 2.1. Materials

A commercially manufactured 125 µm thick PET MELINEX^®^ ST506 from DuPont Teijin Films™ (Dumfries, United Kingdom) film was used as reference (Ref-PET), since PET is a commonly used substrate material in printed electronics applications. MELINEX^®^ ST506 is a clear and heat-stabilised polyester film and has pre-treatment on both sides of its film. The manufacturer claims that film has excellent dimensional stability at temperatures up to 150 °C [38].

Partially bio-based poly(ethylene terephthalate) (Bio-PET) Eastlon PET CB-602AB was purchased from FKuR Kunstoff GmbH (Willich, Germany). It is a multipurpose PET grade comprised of 20% bio-based carbon content, since the second monomer of PET, monoethylene glycol (MEG), is derived from bio-based ethanol. The grade is especially suitable for the production of bottles and films and exhibits good optical clarity. The density of Bio-PET is 1.3–1.4 g/cm^3^.

Poly(lactic acid) Luminy^®^ L175 (PLA) was purchased from Total Corbion Ltd. (Gorinchem, Netherlands). PLA L175 is specified as high-heat grade, and it is 99% optically pure poly(L-lactic acid). It is suitable for film extrusion, thermoforming and fibre spinning. The density of PLA L175 is 1.24 g/cm^3^ and it has a melt flow index (MFI) of 8 g/10 min at 210 °C. Melting of this PLA grade occurs at 175 °C.

Cellulose acetate propionate (CAP), Cellidor^®^ CP 300-13, was purchased from Albis Plastics (Hamburg, Germany). The cellulose raw materials for Cellidor^®^ CAP are drawn from sustainable and natural resources. According to its manufacturer, Cellidor is an amorphous polymer and has a high degree of light transmission. It also has high impact strength even at temperatures below freezing. The material contains 13% phthalate-free plasticiser as an additive. CAP has a heat deflection temperature of 85 °C and density of 1.2 g/cm^3^.

NatureFlex™ 30 NVO is a commercial regenerated cellulose film with a thickness of 30 µm, from Futamura Chemical Co. Ltd (Nagoya, Japan). It is derived from renewable resources (bio-based carbon content 96%) and is certified as compostable in industrial and home composting environments. The manufacturer states that the NatureFlex™ is suitable for printing and for lamination to other biopolymers. The principal raw material for the NatureFlex™ is cellulose derived from wood pulp. Natureflex film has heat-seal coating on both sides of the film. The coating material was not disclosed by the manufacturer [34].

### 2.2. Cast Film Extrusion

Materials were dried prior to processing. CAP and PLA were dried overnight at 65 °C in a vacuum oven. Bio-PET was first dried at 150 °C in an oven, after which the granules were put into a vacuum chamber for three hours. Films were manufactured with a laboratory-scale single-screw extruder Brabender Plastograph EC plus 19/25 D (Brabender GmbH & Co. KG, Duisburg, Germany). The sheet die was 120 mm wide, and the films were cast on heated roll stack to avoid heat-shock. To avoid excess moisture absorption, a nitrogen stream of 5 L/min was fed into the feeder. Screw geometry was conical with a compression ratio of 3:1. A sieve of 40/80 mesh was used in the extruder. Table 1 shows processing parameters and film thicknesses for Bio-PET, PLA, and CAP.

### 2.3. Orientation and Annealing

Cast films were oriented to make thinner films and to attain oriented microstructure. In the case of the semi-crystalline polyesters, PET and PLA, orientation improves crystallinity via strain-induced crystallisation [39]. Orientation was carried out biaxially (BO) with a Brückner Karo IV laboratory-scale stretcher (Brückner Maschinenbau GmbH & Co. KG, Siegsdorf, Germany). Orientation parameters were tailored for each material so that the maximum orientation ratio could be achieved without film tearing. Biaxial stretching was executed in simultaneous mode. Samples were cut into 8.5 cm × 8.5 cm squares and inserted into stretcher clips. The applied clip pressure was 40–50 bar depending on how thick the films were. During the orientation process, the films were first pre-heated for a defined time, after which stretching occurred at a pre-defined stretch rate.

In case of CAP, the orientation parameters were experimentally defined, as there was not much information about its orientation behaviour. For polyesters such as PET and PLA a suitable orientation temperature can usually be found 15–20 °C above their glass transition temperature. Table 2 shows the applied orientation parameters for each material.

Annealing was performed in a custom-made annealing frame. The purpose of the frame was to keep the oriented film in tension and thereby prevent shrinkage during heat treatment. The annealing frame was adjustable so that the free area in the middle of the frame was as large as possible for films having different orientation ratios. Annealing temperatures were chosen, based on the orientation temperatures and each material’s melting temperature, so that the annealing was carried out below *T_m_*. The annealing time for each material was four minutes and it was carried out in an oven. The annealing Temperature for Bio-PET was 225 °C, for PLA 140 °C, and for CAP 110 °C.

### 2.4. Thermal Analysis

Cast films were examined with differential scanning calorimetry (DSC) to observe the transition temperatures and crystallinities of materials. Transitions such as glass transition (*T_g_*) and melting temperature (*T_m_*) are important in evaluating the heat resistance of materials. Further, the development of crystallinity due to orientation and annealing were studied with differential scanning calorimetry in the case of semi-crystalline materials.

During DSC measurements, heating and cooling runs were carried out twice at a rate of 10 K/min. The temperature range was slightly different for some of the materials depending on their expected melting temperature and glass transition temperature.

The crystallinity of semi-crystalline polymers can be calculated as follows.

(1) $$X_{c}=\frac{\Delta H_{m}-\Delta H_{cc}}{\Delta H_{m}^{{}^{\circ}}}\times 100\%$$

where $\Delta H_{m}$ is the melting enthalpy (J/g), $\Delta H_{cc}$ is the enthalpy of cold crystallisation (J/g) and $\Delta H_{m}^{{}^{\circ}}$ is the melting enthalpy of 100% crystalline polymer. Enthalpies of melting for 100% crystalline material can be found in the literature for PET and PLA and they are listed in Table 3 below.

### 2.5. Tensile Properties

Tensile testing for films was performed according to SFS-EN ISO 527-1 and SFS-EN ISO 527-3 with the Instron 4505 universal material tester (Instron Corp., Norwood, MA, USA) with 1000 N load cell and the Instron 2665 Series High Resolution Digital Automatic Extensometer (Instron Corp., Norwood, MA, USA). Tests were performed with a 5 mm/min extension rate. Tests were performed at standard conditions (23 °C and 50% RH). The specimen geometry was type 2. The initial distance between grips was 100 mm with gauge length of 50 mm for un-oriented samples. Some of the oriented and annealed samples were too small for a 100 mm gripping distance, thus the distance was adjusted so that those specimens could be tested.

### 2.6. Screen-Printing of Silver

Asahi LS 411 AW silver paste (Asahi Chemical Research Laboratory Co., Ltd., Tokyo, Japan) was printed with sheet-based flat-bed screen-printing equipment (EKRA XH STS, ASYS Group GmbH, Boennigheim, Germany) using 325 L mesh screen with 15 mm emulsion and 28 mm wire (Murakami, Tokyo, Japan). The silver was printed using a 1.2 mm snap off distance, a 0.2 mm down stop, 70 N pressure, and a 30 mm/s printing speed and dried in an oven (circulating heat) under 120 °C (Ref-PET), 100 °C (Bio-PET) and 80 °C (CAP, Natureflex, and PLA) for 30 min.

### 2.7. Hybrid-Integration of LEDs

Red, green and yellow WL-SMCC SMT Mono-color Chip LED Compact (Würth Electronics eiSos GmBH Co. & KG, Waldenburg, Germany) and white ASMT-CW20 SMT 0.2 mm Top Fire Chip LED (AVAGO Technologies Ltd., San Jose, CA, USA) were bonded on silver printed PET, PLA, and CAP films with EpoTek H20E (Epoxy Technology, Inc., Billerica, MA, USA) and cured at 80 °C for three hours. UV-curing adhesive Dymax 9008 (Dymax Corporation, Torrington, CT, USA) was dispensed around the LEDs to improve the mechanical support.

### 2.8. Film Characterisation

The film thickness was measured with an optical profilometer (Vantage Controller 50, cyberTECHNOLOGIES GmbH, Eching-Dietersheim, Germany) and optical transmission with a UV–Vis–NIR spectrophotometer (Cary 5000, Agilent, Santa Clara, CA, USA). The surface roughness was measured with a white light interferometer (Wyko NT3300, Veeco, Plainview, NY, USA) in vertical scanning interferometry (VSI) mode with 20.8× magnification and plane fit (tilt). Arithmetic roughness (Ra was calculated by fitting the absolute surface profile data with the mean surface level, root-mean-squared roughness (Rq) by fitting the root-mean-squared data from the surface profile with the mean surface level, and total roughness height (Rt) from the peak-to-valley differences. Prior to the measurement, a 60 nm silver layer was thermally vacuum evaporated (MB 200B, MBRAUN, Munich, Germany).

The accuracy of printed patterns was measured with a microscope (Smartscope OGP250, Optical Gaging Products, Rochester, NY, USA), the printed silver thickness with an optical profilometer (Vantage Controller 50, cyberTECHNOLOGIES GmbH, Eching-Dietersheim, Germany) and the resistance with a Multimeter (189 True RMS Multimeter, Fluke, Everett, WA, USA). Surface energy measurements were obtained using a contact angle meter (CAM200, Biolin Scientific, Stockholm, Sweden), and measured using water, ethylene glycol, and diiodomethane. Surface energy calculations were performed according to the Fowkes’ Geometric Mean method.

### 2.9. Scanning Electron Microscopy (SEM)

Scanning electron microscopy (SEM) images were taken from cross-sections of the S2S screen-printed polymer films to study adhesion between the silver paste and film surface. The equipment was JEOL JSM-6360LV SEM (JEOL Ltd., Tokyo, Japan) with a tungsten hairpin filament as an electron source. Imaging was performed with secondary electron signals (SE). Prior to SEM imaging, cross-sections were prepared by breaking samples in liquid nitrogen immersion to ensure a brittle fracture without plastic deformation. Samples were gold sputtered for 130 s with a Baltec Balzers (Balzers Union, Vaduz, Liechtenstein) sputter coater. Images were taken with acceleration voltage of 8 kV and a spot size of 34. Working distance was between 10 and 16 mm.

## 3. Results and Discussion

### 3.1. Thermal Properties and Crystallinity

Thermal properties, including glass transition (*T_g_*), cold crystallisation (*T_cc_*), melting (*T_m_*), and melt crystallisation (*T_mc_*) temperatures, of the analysed film samples are presented in Table 4.

From Table 4, Ref-PET shows the highest melting temperature at 254 °C of all of the tested materials. Bio-PET has a slightly lower melting temperature at 249 °C than the commercial Ref-PET. Orientation and annealing increased the glass transition temperature of Bio-PET from 72.3 °C to 80.5 °C, which is most likely a result of increased crystallinity and thereby restricted chain mobility in an amorphous phase. Both PET grades show melt-crystallisation behaviour upon cooling. Melt crystallisation of Ref-PET occurs at 196 °C while Bio-PET shows melt-crystallisation at temperatures below 190 °C.

Similar to Bio-PET, the glass transition of PLA also increased as a consequence of orientation and annealing (Table 4). Unoriented PLA has a *T_g_* of 60.3 °C, while the oriented and annealed PLA is 65.7 °C. Melting of PLA occurs around 176 °C and is not affected by orientation or annealing. However, annealing causes the disappearance of the cold crystallization peak due to highly crystalline structure, as presented in Table 5.

Presented in Table 4, CAP has a glass transition around 100 °C before annealing. After annealing, the glass transition seems to increase to 109.5 °C. There is also a minor melting peak at 155–163 °C, however, it does not have well-defined shape such as semi-crystalline polymers, and the integrated enthalpies are less than 16 J/g.

Natureflex has a *T_g_* of 68 °C and clear melting peak of 179 °C. Natureflex is a regenerated cellulose film which is not a thermoplastic material, thereby the melting peak is most likely connected to the thermoplastic heat-seal coating on both sides of the Natureflex film.

The transition enthalpies and crystallinities of semi-crystalline polymers Ref-PET, Bio-PET, and PLA are presented in Table 5. Natureflex is excluded from this table, since the observed melting enthalpy describes the thermal behaviour of the heat-seal coating, not the Natureflex film itself.

Table 5 shows that Ref-PET has higher crystallinity at 44.7% than oriented and annealed Bio-PET, which has crystallinity of 33.3%. PLA has crystallinity of 49% after orientation and annealing. Biaxial orientation of the Bio-PET and PLA cast films is an important step toward achieving crystalline structure. Unoriented PLA exhibits amorphous morphology and unoriented Bio-PET has low crystallinity of 6.5%. Crystalline structure is known to improve mechanical performance and thermal stability of semi-crystalline polymers [41].

### 3.2. Tensile Properties

**Figure 1:**
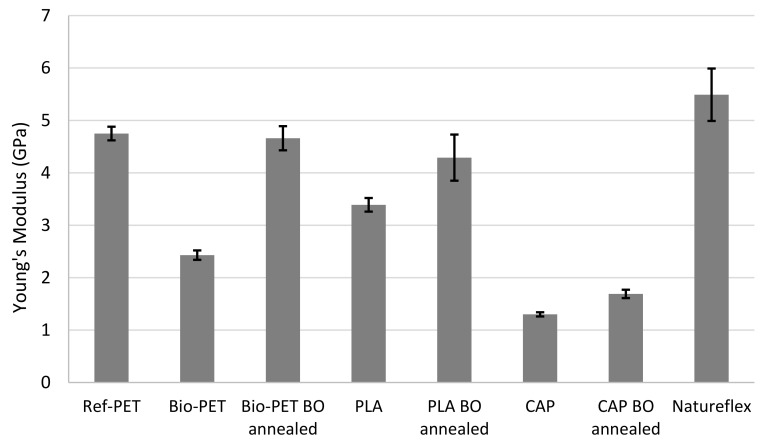
Young’s modulus values for film materials.

**Figure 2:**
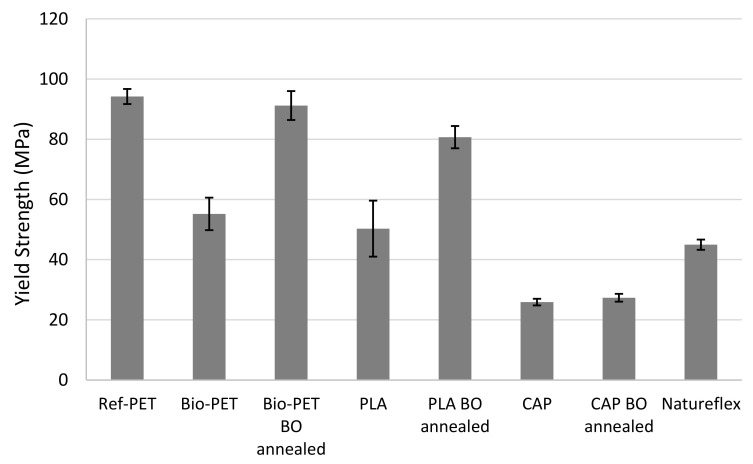
Yield strength of analysed film materials.

**Figure 3:**
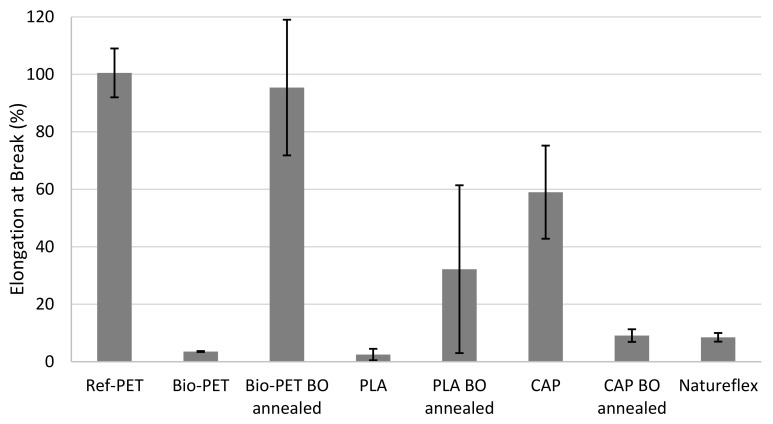
Elongation at break (%) values.

The tensile strength properties, including the Young’s modulus, yield strength, and elongation at break values, for the films are presented in Table 6. The results are presented graphically in Figure 1, Figure 2 and Figure 3. Figure 1 shows the Young’s modulus values of tested materials, Figure 2 presents a visual comparison of yield strengths and Figure 3 compares measured elongation at break values.

Presented in Table 6 and Figure 1, the highest modulus value (5.49 GPa) is achieved with the commercial Natureflex film. Bio-PET and commercial reference PET also exhibit high modulus values. As a result of biaxial orientation, an annealed Bio-PET has 92% higher modulus than unoriented Bio-PET. The modulus of the oriented Bio-PET is 4.66 GPa, whereas the un-oriented Bio-PET has a modulus of 2.43 GPa. Stiffness of the oriented and annealed Bio-PET is similar to the commercial Ref-PET, which has modulus of 4.75 GPa. Similar results have been reported by Bandla et al., in their study the unoriented PET film has a modulus of 4.3 GPa while biaxial orientation results in modulus of 4.7 GPa [16].

As presented in Table 6 and Figure 1, orientation and annealing also improved the PLA stiffness: the unoriented PLA has modulus of 3.39 GPa while the biaxially oriented and annealed PLA has 4.29 GPa. When comparing stiffness properties, CAP exhibits lower modulus values than other films. Unoriented CAP film has a modulus of 1.30 GPa, while the biaxially oriented and annealed CAP has 1.69 GPa.

Presented in Table 6 and Figure 2, the highest yield strengths are obtained with the oriented Bio-PET and commercial Ref-PET. Biaxially oriented Bio-PET has a yield strength of 91 MPa and Ref-PET has 94 MPa. Orientation of Bio-PET results in a 65% higher yield strength. A similar trend is observed with the PLA, wherein orientation improves yield strength by 60%, resulting in a yield strength of 80 MPa. In the case of CAP, orientation and annealing has less dramatic impact on the yield strength; the yield strength of both unoriented and oriented CAP is in the range of 25–27 MPa. Natureflex has a lower yield strength than the PET grades or PLA at 45 MPa.

As presented in Table 6 and Figure 3, the unoriented PET and PLA are brittle with elongation at break values less than 5%. Orientation and annealing improved their ductility in comparison to the untreated films. The oriented and annealed Bio-PET has elongation of 95% which is similar to the Ref-PET, which has elongation at break of 100%. The oriented and annealed PLA has elongation at break of 32%. In the case of the CAP, orientation and annealing decreased elongation at break values by 85%. The Natureflex film is relatively brittle, with elongation at break value of 8.5%.

### 3.3. Transparency and Surface Properties

**Figure 4:**
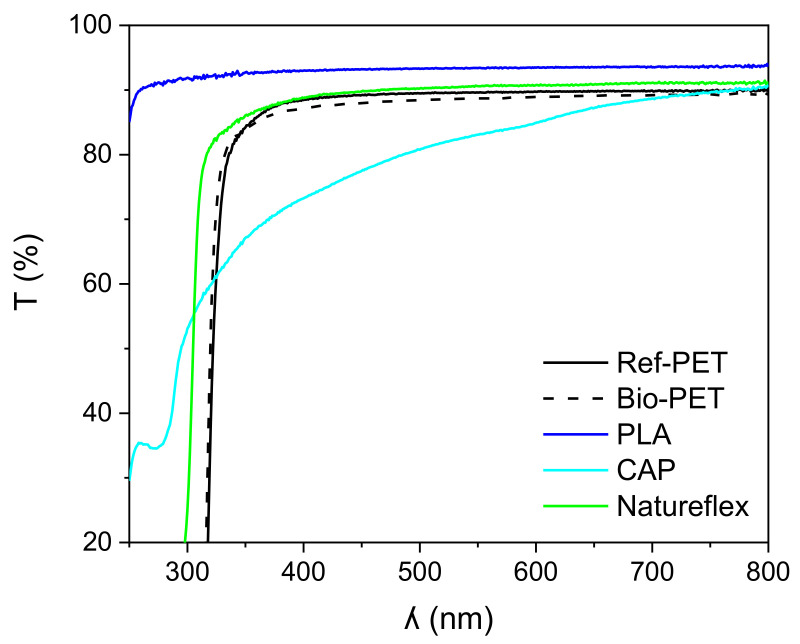
Optical transmission (T) of the Ref-PET, Bio-PET, PLA, CAP and Natureflex films versus wavelength (λ).

**Figure 5:**
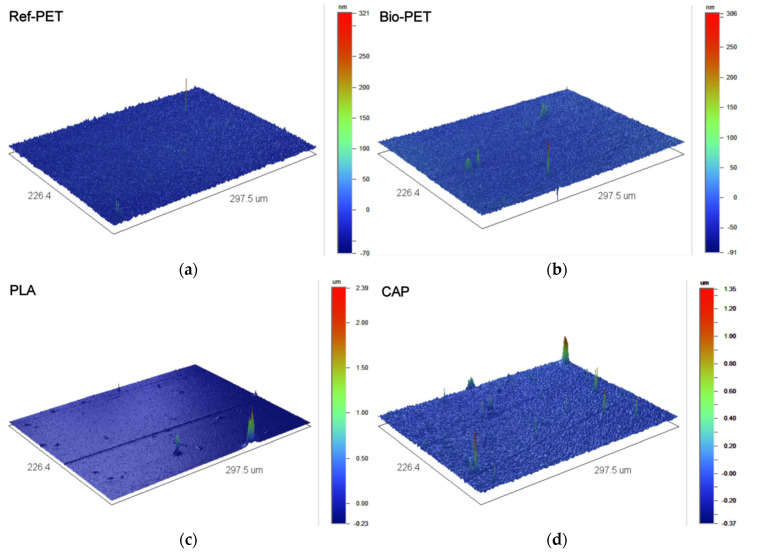

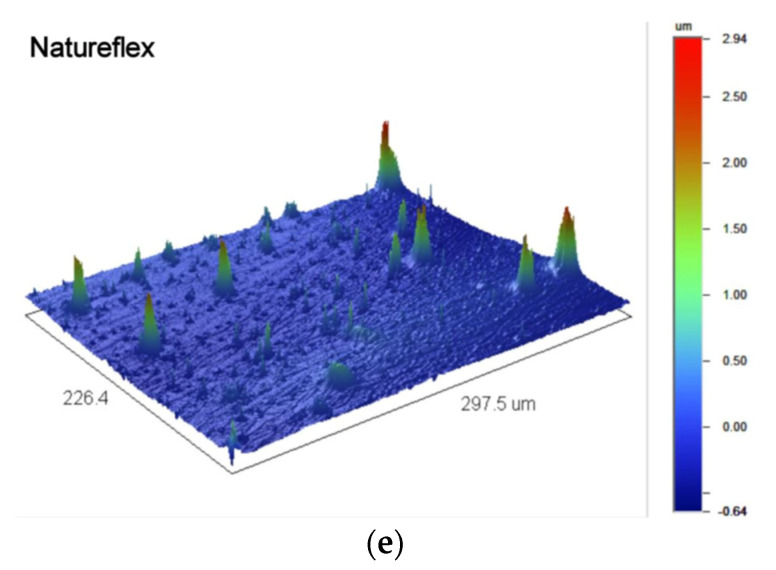
Surface profile of (**a**) Ref-PET; (**b**) Bio-PET; (**c**) PLA; (**d**) CAP; and (**e**) Natureflex film using white light interferometer with a magnitude of 20.8×.

The optical transmission, surface roughness, surface topography, and surface energy of the Ref-PET, Bio-PET, PLA, CAP, and Natureflex are presented in Figure 4 and Figure 5, and Table 7 and Table 8.

Figure 4 and Table 7 show that, in comparison to the Ref-PET, the optical transmission of PLA and Natureflex was higher, being between 90 and 94% at 500 nm and 700 nm, respectively, and the optical transmission of the Bio-PET film was similar to the Ref-PET, being between 88 and 89%. The CAP film exhibited the lowest transmission, due to the milky appearance of the film, and the transmission was between 81 and 89% at 500 nm and 700 nm. However, it should be noted that the film thickness values were not the same and the film thickness ranged between 30 µm and 170 µm, thus the small differences in the transmission at a visible region can be explained by the film thickness.

Figure 4 plots the optical transmission versus the wavelength for the films in this study. The transmission of the Bio-PET and Natureflex films were comparable to the Ref-PET film between 350 and 800 nm wavelengths, and higher with PLA. The transmission of the PET, Bio-PET, and Natureflex films rapidly decreased at 310–330 nm, and at 250 nm, the transmission was 1%, whereas the transmission of PLA film was 85–92% between 250 and 350 nm.

Film thickness values were comparable to Ref-PET with an exception of the 30 µm thin Natureflex, which made it more challenging to handle. From Table 7 and Figure 5, the surface of the Bio-PET film was comparable to Ref-PET in terms of the surface roughness (R_a_, R_q_ and R_t_) and topology. The surface roughness of PLA and CAP was an order of magnitude higher, while Natureflex was two orders of magnitude higher. Heat-seal coating on the surface of the Natureflex film provides the main explanation for the higher surface roughness (Figure 5). Here, the thickness of screen-printed silver is 20 µm, thus, the roughness values presented in Table 7 are acceptable for this work. If the electronic structures comprise thinner layers and the material thickness would range from a few tens of nm to 1 µm, the roughness of Natureflex would be too high.

Table 8 shows results from surface energy measurements for the analysed materials.

In printing and coating, the wetting and adhesion is associated with the surface tension of the ink and the surface energy of the substrate. In general, a proper bonding can be achieved when the surface energy is between 2 to 10 mN/m higher than the surface tension although that is not always the only determinant factor. Problems in the adhesion or wetting are less common if the solvent-based inks or pastes comprise low surface tension solvents, such as ethyl alcohol (22.1 mN/m) and ethyl acetate (24.4 mN/m) [42].

According to the Fowkes model, the total surface energy of the PET, Bio-PET, PLA, CAP, and Natureflex substrates ranged from 38 to 44 mN/m (Table 8), with the dispersive part of the surface tension varying from 31 to 38 mN/m and the polar part from 1 to 10 mN/m. Asahi LS 411 AW is ethyl carbitol acetate-based screen-printing paste that has a surface tension of 31.1 mN/m. In that respect, the surface tension of the printing paste solvent is 6–13 mN/m lower than that of the surface energy of the substrate, and supports proper wetting and adhesion of the ink [42].

Good adhesion is mainly associated with the polar interactions between the substrate and the ink, the polar part of the surface energy can be increased e.g., with plasma treatment [42,43]. Corona treatment and adhesion primers are also possible [42]. In this work, reported in Table 8, the polar part of the surface energy for CAP and PLA substrates was comparable with the Ref-PET, and significantly lower with the Bio-PET and Natureflex. The surface energy data predicted good adhesion strength for the Ref-PET, CAP, and PLA substrates, and lower adhesion strength for Bio-PET and Natureflex. However, the heat-seal coating on Natureflex film is expected to enhance the adhesion [34].

### 3.4. Dimensional Stability and Resistance of S2S Screen-Printed Films

**Figure 6:**
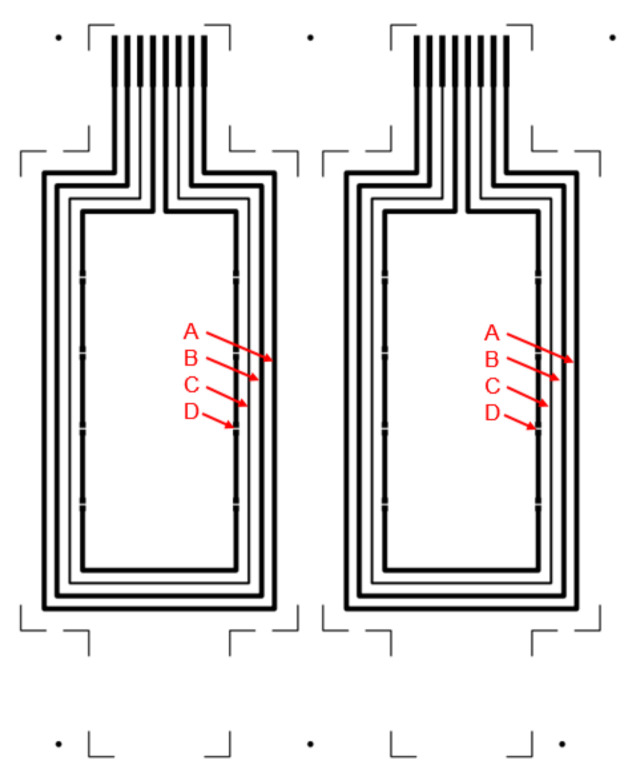
Test layout for S2S screen-printing of silver for comprising two test structures with 1.0 mm line width patterns (outer loop A and B) and 0.5 mm line width (loop C) for characterisation and loop D (inner loop) for LED integration.

**Figure 7:**
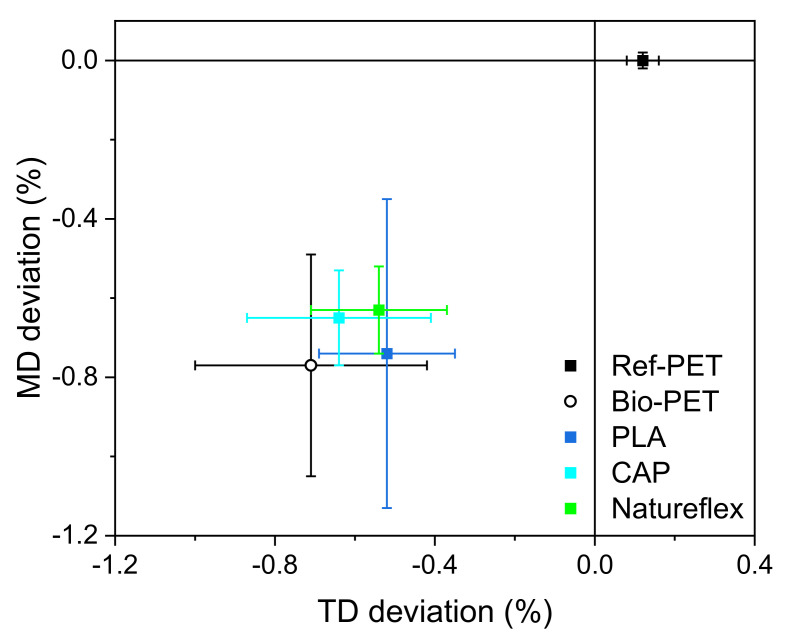
Machine direction (MD) and transverse direction (TD) accuracy of S2S screen-printed LS 411 AW silver patterns compared to the dimensions of the layout.

Figure 6 presents the test layout for S2S screen-printing of silver paste, and Table 9 and Figure 7 present the dimensional accuracies of S2S screen-printed LS 411 AW silver on substrate film.

Temperatures for the thermal curing of the printed silver were evaluated with pre-tests. Higher temperatures can provide better electrical properties; thus, the aim was to have as high a processing temperature as possible. Based on pre-test results, the Ref-PET was processed at 120 °C, Bio-PET at 100 °C and PLA, CAP, and Natureflex at 80 °C for 30 min. Table 9 shows that the screen-printed LS 411 AW pattern obtained the dimensions of the layout in MD on the Ref-PET substrate, and were reduced between 0.6 and 0.8% on the Bio-PET, PLA, CAP and Natureflex. The printed LS 411 AW pattern was increased in TD on Ref-PET 0.1% and decreased between 0.5 and 0.8% on Bio-PET, PLA, CAP and Natureflex, respectively. Within biofilms, the best dimensional accuracy and smallest min–max deviation was obtained when LS 411 AW was printed on Natureflex, thus enabling the scale-up of the dimensional changes and improved reproducibility. Furthermore, the processing at 80 °C would likely increase the dimensional accuracy of the Bio-PET. It should also be noted that, in comparison to R2R processing, the unrestrained film was prone to deform during the S2S thermal treatment, whereas R2R processing provided the possibility of controlling the web and improving dimensional accuracy [5].

**Figure 8:**
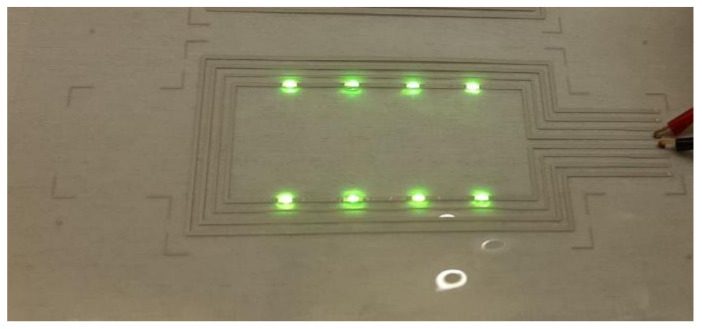
Screen-printed hybrid-integrated LED foil.

Table 10 presents the resistance measurements of S2S screen-printed LS 411 AW silver on substrate films using the test pattern presented in Figure 6, and the functionality of the printed structures was demonstrated with the hybrid-integration of LEDs presented in Figure 8.

In comparison to the 7.0 Ω resistance of the 1.0 mm-wide screen-printed silver pattern on Ref-PET, the silver printing on CAP and Natureflex resulted in resistance of 5.7 Ω and 6.3 Ω which was a significantly lower value and notably it was obtained by using 80 °C for the thermal curing of the silver. The resistance of silver on the Bio-PET and PLA comprised higher resistance. To understand the printed structure, the silver-substrate interface, and the possible correlation with the resistance values, the printed films were analysed with SEM.

### 3.5. SEM Analysis for S2S Screen-Printed Films

**Figure 9:**
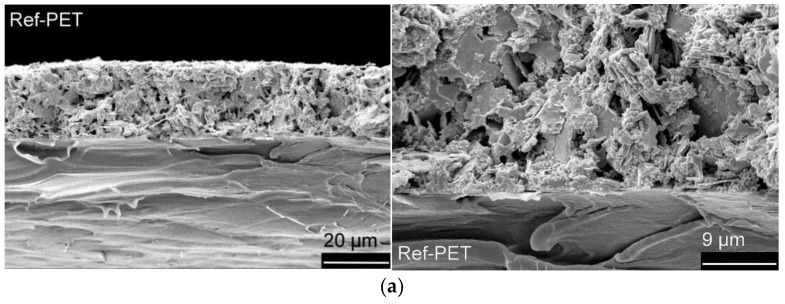

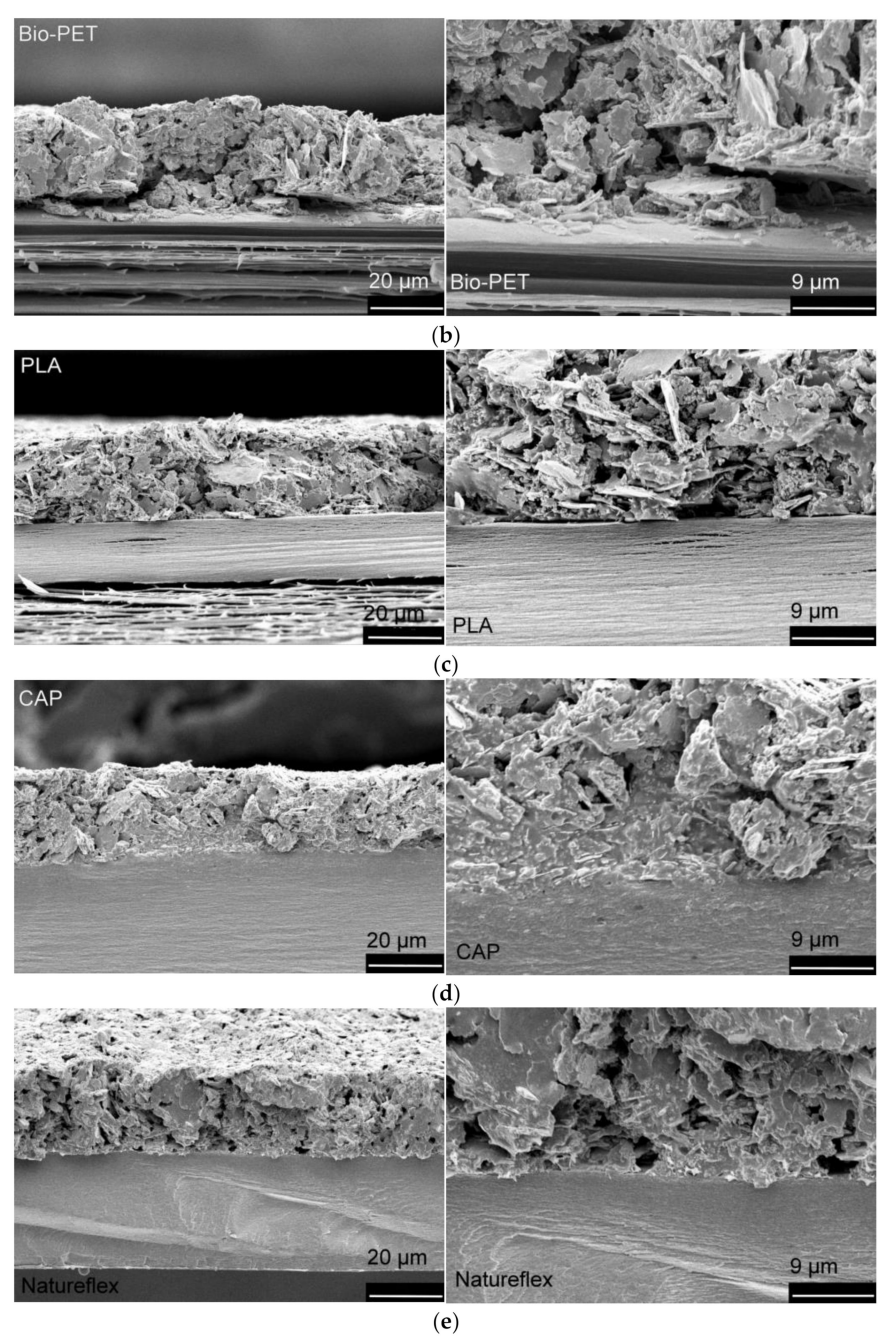
Cross-sectional SEM images of screen-printed LS 411 AW silver on (**a**) Ref-PET; (**b**) Bio-PET; (**c**) PLA; (**d**) CAP; and (**e**) Natureflex films. The images on top are magnified 1000× and the images on below are magnified 2500×.

Cross-sectional SEM images of screen-printed LS 411 AW silver and substrate film interfaces are presented in Figure 9.

The cross-sectional image from the Ref-PET film depicts the reference point. As presented in Section 3.3, the higher polar part in the surface energy of the Ref-PET (Table 8) increases the polar interactions between the printed silver and the substrate promoting the adhesion. The cross-sectional image from the Bio-PET (Figure 9b) shows inadequate contact between the printed silver and the Bio-PET surface. In addition, the printed silver contains cracks that weaken the uniformity of the printed pattern, which can explain the higher resistance presented in Table 10. The poor contact between the printed silver and the substrate can be attributed to the lower polar part of the surface energy (Table 8), as well as to the limited thermal stability of the substrate (Table 9).

The cross-sectional SEM image from the PLA (Figure 9c) reveals that there is a slight gap between the printed silver and the PLA. although the higher polar part in the surface energy of the PLA (Table 8) enhances the adhesion properties. In comparison to the silver-PET, the higher printed silver resistance can be explained with the inadequate silver–PLA contact (Table 10). A cross-sectional SEM image from the CAP (Figure 9d) shows that the adhesion between the printed silver and the CAP is excellent; there are no cracks or cavities in the interface. In comparison to the Ref-PET, the silver-CAP contact is significantly better, and the printed silver resistance is the lowest. In addition to the high polar part of the surface energy, the compatibility is supported by the chemical similarity of CAP and acetate-based printing paste. Similar to the CAP, the cross-sectional image from the Natureflex (Figure 9e) exhibits a good printed silver–Natureflex contact and low printed silver resistance, although the polar part of the surface energy is low. Notably, the heat-seal coating on top of the Natureflex film will likely melt during the thermal curing and improve the printed silver–Natureflex adhesion.

## 4. Conclusions

The aim of this study was to assess the compatibility of various types of bio-based polymer substrates for printed hybrid electronics applications. The main objective was to find more sustainable substrate film materials to replace the fossil-based substrates such as poly(ethylene terephthalate) (PET), without compromising the performance. Very few research articles exist concerning the use of bio-based materials in thin film flexible electronics applications. This study fills a gap in the knowledge and combines screen-printing processing and material development in a novel way.

Three commercial bio-based polymer resins, bio-based poly(ethylene terephthalate) (Bio-PET), poly(lactic acid) (PLA) and cellulose acetate propionate (CAP), were used to manufacture cast-extruded substrate films on a laboratory-scale. In addition, the commercially manufactured bio-based regenerated cellulose film, Natureflex, was investigated. A commercial fossil-based PET film was used as a reference material. Laboratory- scale manufactured films were oriented and annealed to obtain oriented microstructure and improved heat stability via strain-induced crystallization.

Results show that bio-based materials can exhibit similar or even higher performance over the commercial fossil PET film (Ref-PET), even under lower printing process temperatures than the typically used 120 °C. Furthermore, the optical transmission and surface properties were comparable to the reference material. For example, the PLA films had even higher optical transmission than the commercial fossil-based PET-film. The compatibility in surface chemistries also supports the use of bio-based substrates in printed and hybrid electronics, although the limited thermal stability caused some challenges with the PLA and Bio-PET films in selected optimal printing process temperatures.

Screen-printed silver patterns on the CAP and Natureflex films had 18% and 11% lower resistance values than on the commercial PET film, providing better electrical properties due to more uniform and compact structures in the printed patterns. Scanning electron microscopy images showed that the contact between the screen-printed silver and substrate was better with the CAP and Natureflex films. The CAP and Natureflex films had lower dimensional changes during the printing process than the PLA and Bio-PET films, indicating better heat stability.

Surface energies of the substrate films were analysed, assuming correlation to printability and better ink wetting and adhesion. However, thermal stability during the printing process had a significant effect on final formation of ink layer structure. This proves that the surface energies are not enough alone to predict the electrical performance of novel substrates.

Optimization of the bio-based substrates through film orientation and annealing can improve the thermal stability and the surface properties, with limitations caused by the material properties being taken into account in electronics’ processing. Importantly, good electrical performance endorses the use of bio-based substrates. In terms of mechanical performance, the biaxially oriented PLA and Bio-PET films have similar stiffness and tensile strength as the commercial PET film.

This study promotes the use of bio-based substrate materials, especially biaxially oriented CAP, in flexible electronics without compromising the quality and performance of the film and the printed electronics structures. In applications where stiffness, tensile strength, and optical clarity are emphasized, the biaxially oriented Bio-PET and PLA films have excellent performance. The regenerated cellulose-based film, Natureflex, can also open novel solutions that require biodegradability. For further studies, a detailed surface analysis could be employed to identify the substrate-ink adhesion properties of different material combinations.

## Figures and Tables

**Table 1:** Processing parameters and film thicknesses for Bio-PET, PLA, and CAP.

Material	Screw	RPM	Temperature Profile from Die to Feeder	Chill Roll Temperature (°C)	Film Thickness (μm)
Bio-PET	3:1	50	270–270–270–270–270–270 °C	80	700
PLA	3:1	50	205–205–200–200–200–200 °C	60	650–700
CAP	3:1	50	210–210–210–200–200–200 °C	80	650–700

**Table 2:** Biaxial orientation (BO) parameters for Bio-PET, PLA and CAP films.

Material	Temperature (°C)	Pre-Heating Time (s)	BO Ratio	Stretch Rate (%/s)	Thickness after (μm)
Bio-PET	88	120	2.9 × 2.9	100	80–100
PLA	77	120	2.9 × 2.9	100	80–100
CAP	137	90	2.1 × 2.1	100	150–170

**Table 3:** Enthalpies of melting (Δ*H_m_*) of 100% crystalline polymers found in literature.

Material	$\Delta H_{m}^{{}^{\circ}}(J/g)$
PET [40]	140.1
PLA [41]	93.6

**Table 4:** Glass transition (*T_g_*), cold crystallisation (*T_cc_*), melting (*T_m_*), and melt crystallisation temperatures (*T_mc_*) of different film materials as un-oriented, biaxially oriented (BO) and biaxially oriented and annealed.

Material	*T_g_* (°C)	*T_cc_* (°C)	*T_m_* (°C)	*T_mc_* (°C)
Ref-PET	77.7 ± 0.0		254.1 ± 1.5	195.9 ± 1.2
Bio-PET	72.3 ± 0.0	131.3 ± 0.20	248.8 ± 0.7	186.3 ± 0.4
Bio-PET BO	83.5 ± 0.0	−	249.3 ± 0.1	188.8 ± 0.6
Bio-PET BO annealed	80.5 ± 0.2	−	248.7 ± 0.1	186.8 ± 0.1
PLA	60.3 ± 0.1	107.9 ± 0.1	176.9 ± 0.3	−
PLA BO	66.9 ± 0.1	93.5 ± 3.3	176.5 ± 0.0	−
PLA BO annealed	65.7 ± 0.1	−	175.6 ± 0.3	−
CAP	97.0 ± 1.1	−	155.8 ± 0.1	−
CAP BO	102.1 ± 0.2	−	158.4 ± 1.2	−
CAP BO annealed	109.5 ± 3.5	−	162.7 ± 1.4	−
Natureflex	68.2 ± 0.0	−	178.9 ± 0.0	−

**Table 5:** Melting enthalpies (Δ*H_m_*), crystallisation enthalpies (Δ*H_cc_*) and calculated crystallinities (*X_C_*) of Ref-PET, Bio-PET and PLA films.

Sample	Δ*H_m_* (J/g)	Δ*H_cc_* (J/g)	*X_C_* (%)
Ref-PET	62.7 ± 3.7	−	44.7 ± 2.6
Bio-PET	40.6 ± 1.2	31.4 ± 0.6	6.5 ± 0.4
Bio-PET BO	50.7 ± 0.2	−	36.2 ± 0.1
Bio-PET BO annealed	46.7 ± 0.6	−	33.3 ± 0.4
PLA	45.7 ± 1.4	46.3 ± 0.10	0.0 ± 1.33
PLA BO	61.0 ± 2.4	31.6 ± 1.2	31.4 ± 3.9
PLA BO annealed	48.8 ± 1.0	2.9 ± 1.2	49.0 ± 2.3

**Table 6:** Tensile strength properties of films.

Sample	Young’s Modulus (GPa)	Yield Strength (MPa)	Strain at Break (%)
Ref-PET	4.75 ± 0.13	94.2 ± 2.5	100.5 ± 8.5
Bio-PET	2.43 ± 0.09	55.2 ± 5.4	3.55 ± 0.17
Bio-PET BO annealed	4.66 ± 0.23	91.2 ± 4.8	95.4 ± 23.6
PLA	3.39 ± 0.13	50.3 ± 9.3	2.5 ± 2.0
PLA BO annealed	4.29 ± 0.44	80.7 ± 3.7	32.2 ± 29.2
CAP	1.30 ± 0.04	25.9 ± 1.1	59.0 ± 16.2
CAP BO annealed	1.69 ± 0.08	27.35 ± 1.3	9.1 ± 2.2
Natureflex	5.49 ± 0.50	44.97 ± 1.7	8.5 ± 1.5

**Table 7:** Mean arithmetic roughness (Ra), root-mean-squared roughness (Rq), total height of roughness (Rt) and optical transmission (T), of substrate films.

Substrate	Ra (nm)	Rq (nm)	Rt (µm)	T (%) @500 nm	T (%) @700 nm
Ref-PET	7 ± 2	9 ± 2	0.3 ± 0.1	89	90
Bio-PET BO Annealed	4 ± 0	6 ± 1	0.4 ± 0.1	88	89
PLA BO Annealed	9 ± 1	37 ± 7	2.2 ± 0.5	93	94
CAP BO Annealed	23 ± 7	36 ± 5	1.6 ± 0.1	81	89
Natureflex	105 ± 12	184 ± 20	3.7 ± 0.1	90	91

**Table 8:** Polar and dispersive part of surface energy (SFE) of different substrates according to Fowkes.

Substrate	SFE Dispersive Part (mN/m)	SFE Polar Part (mN/m)	SFE Total (mN/m)
Ref-PET	34.42	9.60	44.02
Bio-PET BO Annealed	38.64	1.76	40.39
PLA BO Annealed	31.34	6.78	38.12
CAP BO Annealed	32.06	7.90	39.96
Natureflex	36.40	0.80	37.20

**Table 9:** Machine direction (MD) and transverse direction (TD) accuracy of S2S screen-printed LS 411 AW silver patterns compared to the dimensions of the layout.

Substrate	Process T (°C)	Mean Dev MD (%)	Max; Min Dev MD (%)	Mean Dev TD (%)	Max; Min Dev TD (%)
Ref-PET	120	0.00	0.02; −0.01	0.12	0.17; 0.07
Bio-PET BO Annealed	100	−0.77	−0.49; −1.18	−0.71	−0.48; −1.10
PLA BO Annealed	80	−0.74	0.07; −1.12	−0.52	−0.34; −0.72
CAP BO Annealed	80	−0.65	−0.50; −0.79	−0.64	−0.10; −1.06
Natureflex	80	−0.63	−0.41; −0.70	−0.54	−0.31; −0.74

**Table 10:** Resistance of S2S screen-printed LS 411 AW silver patterns with 1.0 mm (A, B) and 0.5 mm (C) line width on different substrates.

Substrate	Ag Thickness (µm)	Process T (°C)	Pattern-A Mean R (Ω)	Pattern-B Mean R (Ω)	Pattern-C Mean R (Ω)
Ref-PET	20 ± 2	120	7.2 ± 0.2	7.0 ± 0.2	12.4 ± 0.3
Bio-PET BO Annealed	21 ± 1	100	8.4 ± 0.2	8.3 ± 0.2	14.8 ± 0.6
PLA BO Annealed	23 ± 3	80	8.4 ± 0.6	8.1 ± 0.6	15.2 ± 1.3
CAP BO Annealed	18 ± 2	80	5.9 ± 0.3	5.7 ± 0.2	10.6 ± 0.4
Natureflex	21 ± 1	80	6.4 ± 0.1	6.3 ± 0.1	11.5 ± 0.3

## Data Availability

The authors confirm that the data supporting the findings of this study are available within the article.
